# Hepatitis B Virus and Hepatitis C Virus Affect Mitochondrial Function Through Different Metabolic Pathways, Explaining Virus-Specific Clinical Features of Chronic Hepatitis

**DOI:** 10.1093/infdis/jiae210

**Published:** 2024-04-24

**Authors:** Sakthi Priya Selvamani, Anis Khan, Enoch S E Tay, Matthew Garvey, Harout Ajoyan, Eve Diefenbach, Brian S Gloss, Thomas Tu, Jacob George, Mark W Douglas

**Affiliations:** Storr Liver Centre, The Westmead Institute for Medical Research, The University of Sydney at Westmead Hospital, Westmead, New South Wales, Australia; Storr Liver Centre, The Westmead Institute for Medical Research, The University of Sydney at Westmead Hospital, Westmead, New South Wales, Australia; Storr Liver Centre, The Westmead Institute for Medical Research, The University of Sydney at Westmead Hospital, Westmead, New South Wales, Australia; Storr Liver Centre, The Westmead Institute for Medical Research, The University of Sydney at Westmead Hospital, Westmead, New South Wales, Australia; Storr Liver Centre, The Westmead Institute for Medical Research, The University of Sydney at Westmead Hospital, Westmead, New South Wales, Australia; Protein Core Facility, The Westmead Institute for Medical Research, Westmead, New South Wales, Australia; Westmead Research Hub, The Westmead Institute for Medical Research, Westmead, New South Wales, Australia; Storr Liver Centre, The Westmead Institute for Medical Research, The University of Sydney at Westmead Hospital, Westmead, New South Wales, Australia; Storr Liver Centre, The Westmead Institute for Medical Research, The University of Sydney at Westmead Hospital, Westmead, New South Wales, Australia; Storr Liver Centre, The Westmead Institute for Medical Research, The University of Sydney at Westmead Hospital, Westmead, New South Wales, Australia; Centre for Infectious Diseases and Microbiology, Sydney Infectious Diseases Institute, The University of Sydney at Westmead Hospital, Westmead, New South Wales, Australia

**Keywords:** hepatitis C virus, hepatitis B virus, mitochondria, metabolism

## Abstract

**Background:**

Hepatitis C virus (HCV) and hepatitis B virus (HBV) cause chronic hepatitis with important clinical differences. HCV causes hepatic steatosis and insulin resistance, while HBV confers increased risk of liver cancer. We hypothesized these differences may be due to virus-specific effects on mitochondrial function.

**Methods:**

Seahorse technology was used to investigate effects of virus infection on mitochondrial function. Cell-based assays were used to measure mitochondrial membrane potential and quantify pyruvate and lactate. Mass spectrometry was performed on mitochondria isolated from HBV-expressing, HCV-infected, and control cells cultured with isotope-labelled amino acids, to identify proteins with different abundance. Altered expression of key mitochondrial proteins was confirmed by real-time polymerase chain reaction (PCR) and western blot.

**Results:**

Reduced mitochondrial function and ATP production were observed with HCV infection and HBV expression. HCV impaired glycolysis and fatty acid oxidation, promoting lipid accumulation whereas HBV caused lactate accumulation. In HBV-expressing cells enrichment of pyruvate dehydrogenase kinase inhibited pyruvate to acetyl-CoA conversion thereby reducing its availability for mitochondrial oxidative phosphorylation.

**Conclusions:**

HBV and HCV impair mitochondrial function. HCV infection reduces lipid oxidation causing its accumulation and fatty liver disease. HBV infection affects pyruvate processing causing lactate accumulation, cellular stress, and increased risk of liver disease and cancer.

## Teaser

HBV and HCV impair mitochondrial function. HCV infection reduces breakdown of fat, causing lipid accumulation and fatty liver disease. HBV infection affects pyruvate processing resulting in lactate accumulation, causing cellular stress and increasing the risk of liver disease and cancer.

## Graphical abstract

**Graphical abstract:**
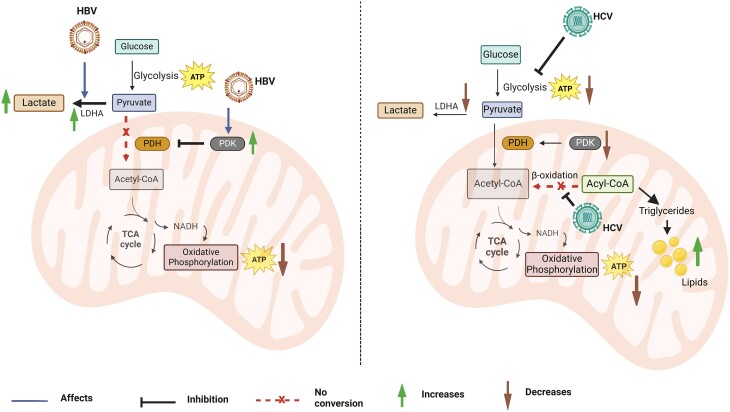
Summary of mitochondrial metabolic regulation by HCV and HBV. This schematic figure summarizes the altered regulation of mitochondrial metabolic pathways during HCV infection and HBV expression. The image was created with BioRender.com.

The World Health Organization estimates 1.4 million people die every year of viral hepatitis complications, predominantly due to hepatitis B virus (HBV) or hepatitis C virus (HCV) [1]. Despite the availability of effective antiviral drugs for both infections, global deaths from viral hepatitis continue to rise [1]. Therefore, a better understanding of the mechanisms leading to viral hepatitis-associated cirrhosis and liver cancer is needed.

A notable difference in chronic liver disease due to HBV and HCV is their contrasting effects on liver metabolism. People with chronic HCV infection have disturbed glucose and lipid metabolism leading to steatosis or insulin resistance and diabetes mellitus, depending on HCV genotype [2, 3]. In contrast, HBV infection is not associated with insulin resistance or steatosis [4] but with reduced prevalence of hypertriglyceridemia and hypercholesterolemia [5]. Another important difference is much higher rates of hepatocellular carcinoma with HBV infection, due in part to persistent oxidative stress [6].

Because of the central role of mitochondria in liver metabolism, we hypothesized that HCV and HBV have different effects on mitochondrial function, explaining the different metabolic phenotypes. In this study we compared the effect of each virus on mitochondrial function in live cells using the Seahorse analyzer, complemented by biochemical, gene expression and proteomics analysis. Our findings provide important insights into the clinical phenotype of each virus and offer the potential for novel therapeutic approaches to prevent and treat chronic liver disease in people with viral hepatitis.

## METHODS

### Cell Culture

For HCV experiments, infection of Huh7 hepatoma cells was established using the JFH1 strain, using published protocols. For HBV experiments the HepG2-derived HepAD38 cell line was used, with standard HepG2s as negative controls. See Supplementary Methods for more details.

### Seahorse Mitochondrial Analysis

To study the effects of HCV and HBV on mitochondrial function, HCV (JFH1) infected Huh7 cells and HepAD38 cells, respectively, were analyzed using the Seahorse analyzer as per the manufacturer's protocol. See Supplementary Methods for more detail.

### Mass Spectrometry and Data Analysis

Stable isotype labeling by amino acids in cell culture (SILAC) analysis was performed to measure the relative abundance of mitochondrial proteins using liquid-chromatography mass spectrometry (LC-MS). See Supplementary Methods for details.

### Statistical Analysis

Statistical analysis of all experiments was carried out using GraphPad Prism 8.0. Unpaired Student *t* test and 1-way ANOVA was applied to calculate the statistical significance. Experiments were considered significant if the *P* value was <.05. For details of Gene Expression Omnibus (GEO) dataset analysis see Supplementary Methods.

## RESULTS

### HCV Infection and HBV Expression Cause Mitochondrial Dysfunction

To study the effects of HCV and HBV on mitochondrial function, Seahorse XF Mito Stress test was performed. This test provides a profile of overall mitochondrial function by measuring oxygen consumption rate (OCR) and also provides information about different parameters of mitochondrial function: basal respiration (baseline OCR required for ATP production); proton leak (remaining basal respiration not coupled to ATP production and dissipated as heat); maximal respiration (maximum rate of respiration by a cell to perform at maximum capacity); spare respiratory capacity (difference between maximal and basal respiration reflecting cell fitness); non-mitochondrial oxygen consumption (OCR by cellular enzymes after addition of rotenone and antimycin A); and ATP-linked respiration (the part of basal respiration required to drive ATP production following injection of oligomycin, an inhibitor of ATP synthase).

For HCV, Huh7 cells infected with HCV (JFH1) and uninfected controls were used. For HBV, as infection rates are relatively low in culture models [7], HepAD38 cells were used, a HepG2-derived cell line that contains an inducible full-length HBV genome controlled by a tet-off promoter. To examine the effect of HBV expression, cells were cultured in the absence of tetracycline. To avoid potential confounding effects of tetracycline on cell metabolism, standard HepG2 cells were used as negative controls. Expression of viral protein in the majority of cells was confirmed for each model (Supplementary Figures 1 and 2).

**Figure 1.**
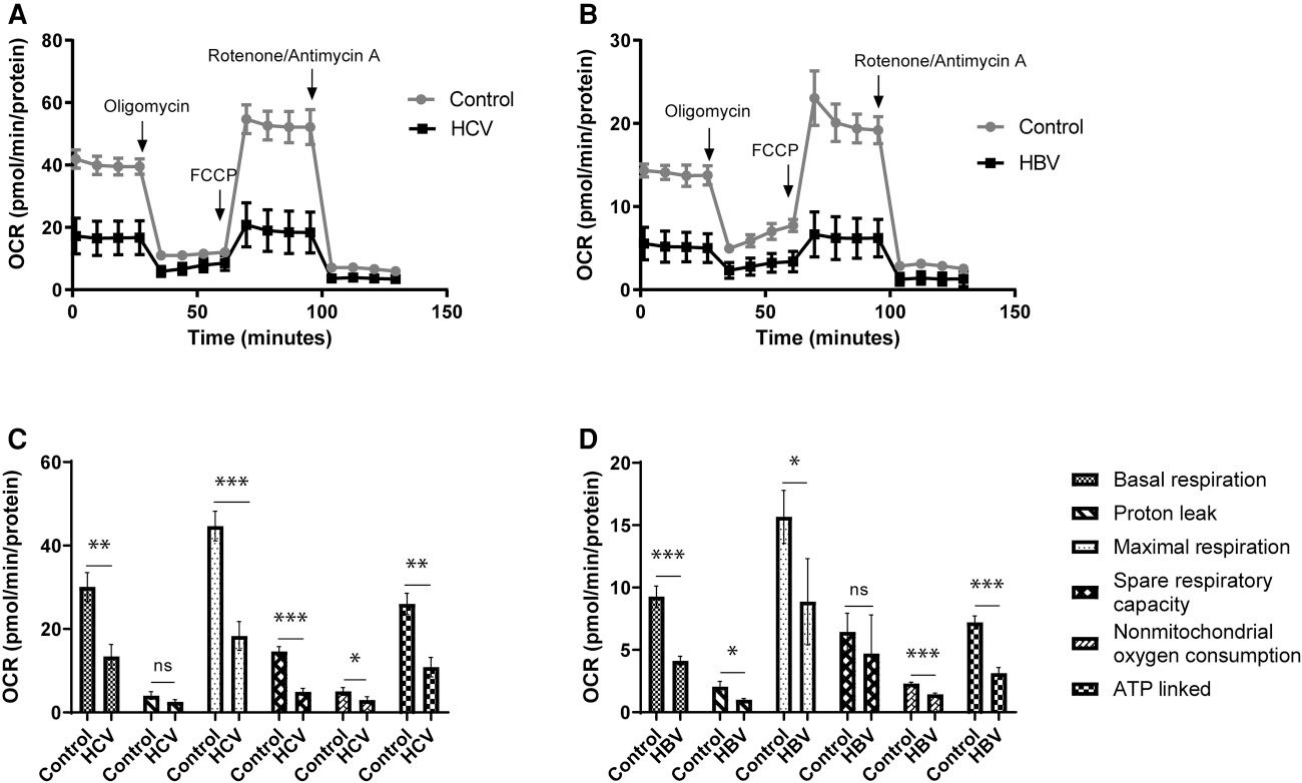
HCV and HBV cause mitochondrial dysfunction. Changes in mitochondrial function in cells infected with HCV (*A*) or HBV (*B*), measured by Seahorse XF Mito Stress Test. Effects of HCV (*C*) or HBV (*D*) on different parameters of mitochondrial function (see key). Each bar represents the average of 3 biological experiments. Error bars represents SEM. *P < 0.05, **P < 0.01, ***P < 0.001. Abbreviations: FCCP, carbonyl cyanide-p-trifluoromethoxyphenylhydrazone; HBV, hepatitis B virus; HCV, hepatitis C virus; ns, not significant; OCR, oxygen consumption rate.

The overall mitochondrial OCR profile was significantly reduced with HCV infection or HBV expression compared to controls (Figure 1*A* and 1*B*, respectively). Both viruses reduced most parameters of mitochondrial OCR, apart from proton leak for HCV (Figure 1*C*) and spare respiratory capacity for HBV (Figure 1*D*).

### HCV and HBV Reduce Mitochondrial ATP Production, But Only HCV Impairs Glycolysis

**Figure 2.**
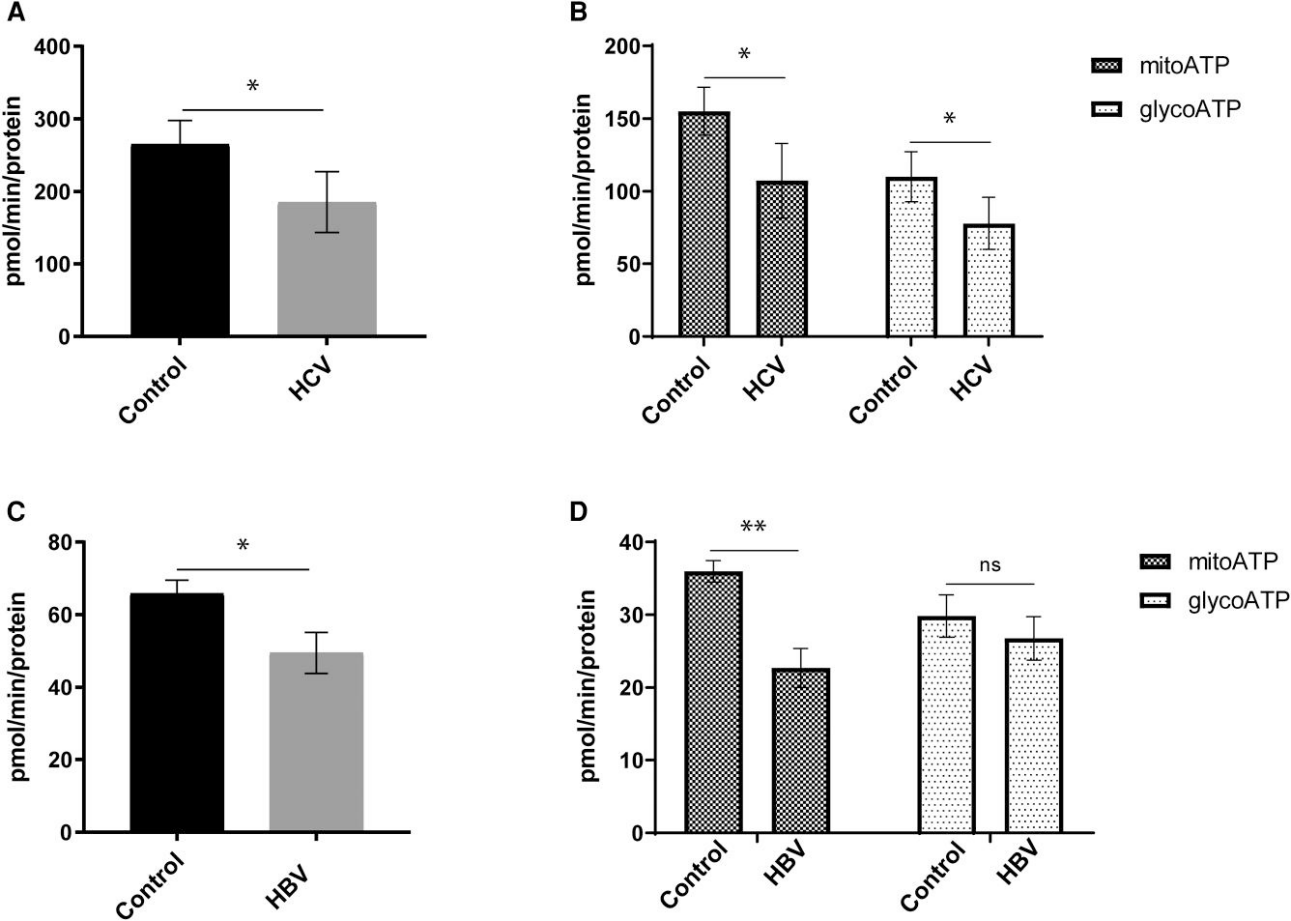
HCV and HBV reduce ATP production. Effects of HCV (*A* and *B*) or HBV (*C* and *D*) on total ATP production and ATP produced from oxidative phosphorylation (mitoATP) and glycolysis (glycoATP) pathways, measured by Seahorse XF Real-Time ATP Rate assay. Each bar represents the average of 3 biological experiments. Error bars represents SEM. *P < 0.05, **P < 0.01. Abbreviations: HBV, hepatitis B virus; HCV, hepatitis C virus; ns, not significant.

To determine the effects of HCV and HBV on ATP production, we used the Seahorse XF ATP rate assay, which measures ATP production rate through two major pathways: glycolysis, which occurs in the cytoplasm; and mitochondrial oxidative phosphorylation (OXPHOS), which occurs in the mitochondria. Total ATP production from both pathways was significantly reduced in HCV-infected (Figure 2*A*) and HBV-expressing cells (Figure 2*C*).

Detailed analysis showed that although mitochondrial ATP production was reduced by both viruses, ATP produced by glycolysis was reduced in HCV-infected cells (Figure 2*B*), but not in HBV-expressing cells (Figure 2*D*). These results suggest that mitochondrial dysfunction impairs ATP production by OXPHOS for both viruses, with additional effects of HCV infection on glycolysis.

### HCV and HBV Decrease Mitochondrial Membrane Potential and Increase Mitochondrial Biogenesis

**Figure 3.**
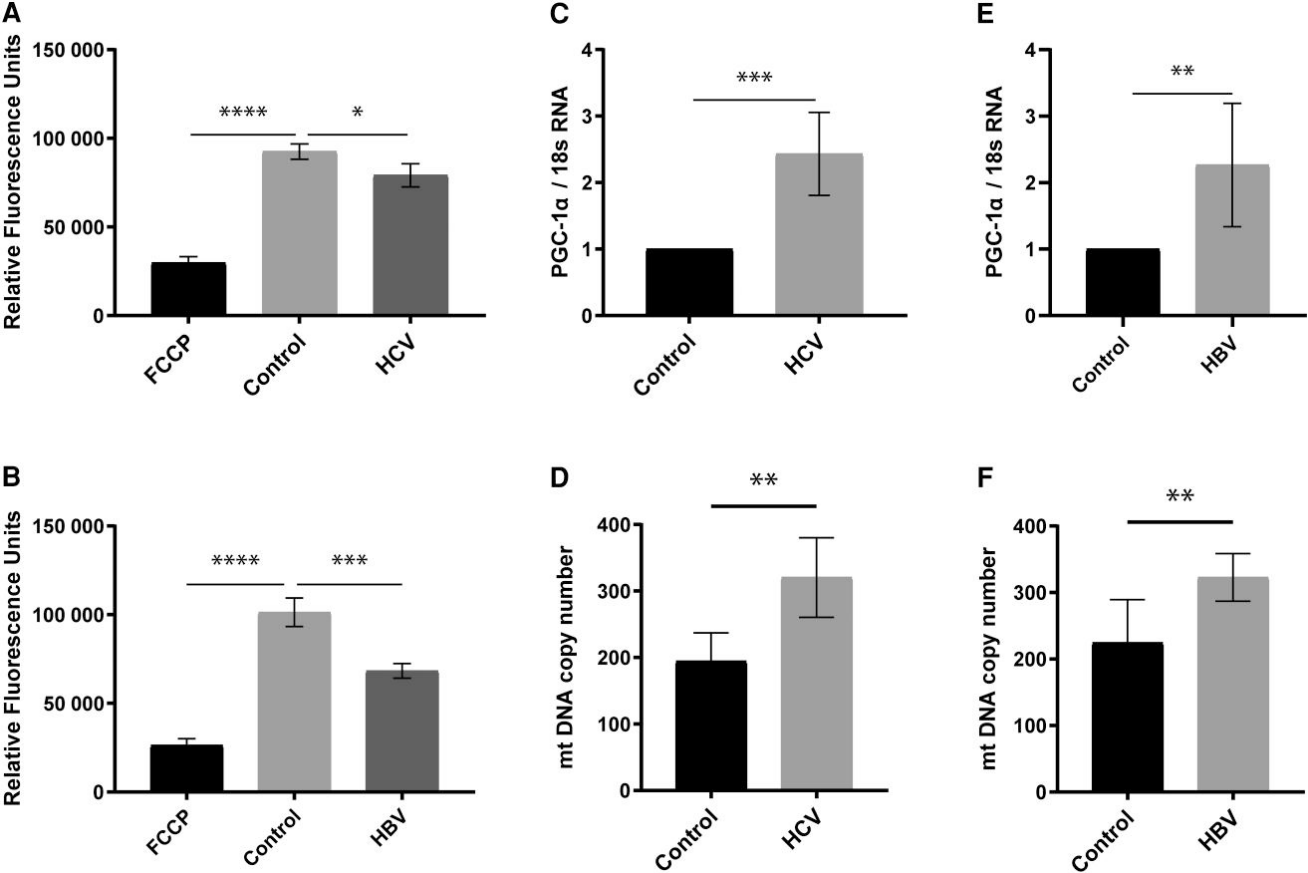
HCV and HBV decrease mitochondrial membrane potential but increase mitochondrial biogenesis. Mitochondrial membrane potential during HCV (*A*) and HBV (*B*) infection was determined by TMRE assay. FCCP treatment served as a positive control. To measure effects of virus infection on mitochondrial biogenesis, PGC-1α mRNA (*C* and *E*) and mitochondrial DNA copy number (*D* and *F*) were determined by real-time PCR. Each bar represents the mean of 3 biological experiments. Error bars represent SEM. *P < 0.05, **P < 0.01, ***P < 0.001, ****P < 0.0001. Abbreviations: HBV, hepatitis B virus; HCV, hepatitis C virus; FCCP, carbonyl cyanide-p-trifluoromethoxyphenylhydrazone; PGC-1α, peroxisome proliferator-activated receptor-γ coactivator-1 α; PCR, polymerase chain reaction.

The mitochondrial membrane potential (ΔΨm) is utilized by complex V (ATP synthase) as an intermediate energy source to drive ATP production [8]. For this experiment we used tetramethyl rhodamine, ethyl ester (TMRE), a cell permeant that accumulates in active mitochondria. Carbonyl cyanide-p-trifluoromethoxyphenylhydrazone (FCCP), an uncoupler of oxidative phosphorylation, was used as a positive control. TMRE fluorescence was reduced in cells infected with HCV (Figure 3*A*) or expressing HBV (Figure 3*B*), demonstrating that both viruses reduce the mitochondrial membrane potential.

Next, we examined the effects of these viruses on mitochondrial biogenesis, to exclude the possibility that reduced mitochondrial function and membrane potential were due to reduced reduced mitochondrial activity, rather than number of mitochondria. Mitochondrial biogenesis was determined by two independent measurements: (1) relative expression of peroxisome proliferator-activated receptor-γ coactivator (PGC-1α); and (2) mitochondrial DNA copy number [9]. We observed increased relative expression of PGC-1α mRNA in cells infected with either HCV (Figure 3*C*) or HBV (Figure 3*E*). Consistent with this, there was increased mitochondrial DNA copy number for both HCV (Figure 3*D*) and HBV (Figure 3*F*). Together, these results indicate that reduced mitochondrial ATP production with both viruses was not due to reduced number of mitochondria.

### HCV But Not HBV Impairs Lipid Metabolism

**Figure 4.**
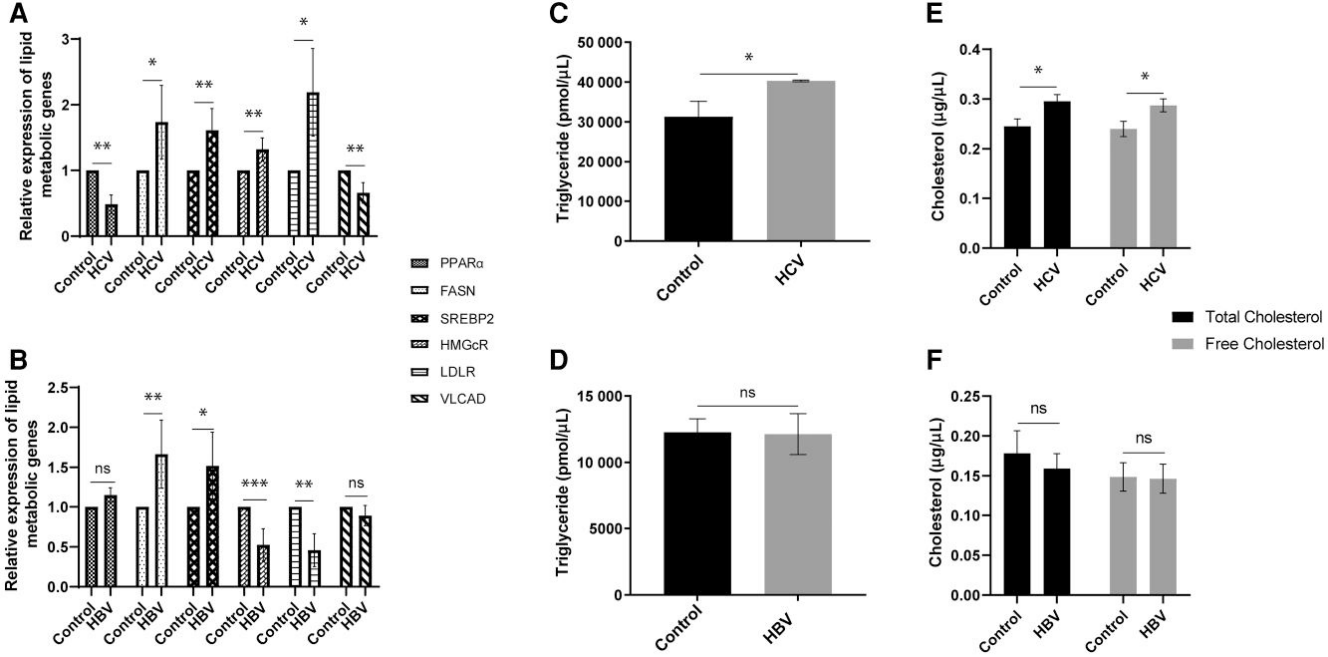
HCV, but not HBV, affects genes involved in lipid metabolism, leading to lipid accumulation. Expression of key genes regulating lipid metabolism was measured during HCV infection (*A*) or HBV expression (*B*) by real-time PCR. Effects of virus on cellular triglyceride (*C* and *D*) and cholesterol (*E* and *F*). Each bar represents the mean of 3 biological experiments. Error bars represent SEM. *P < 0.05, **P < 0.01, ***P < 0.001. Abbreviations: HBV, hepatitis B virus; HCV, hepatitis C virus; ns, not significant; PCR, polymerase chain reaction.

HCV induces steatosis, particularly with genotype 3 infection [2, 10], but HBV infection does not [4, 11]. To examine the effects of virus infection on triglyceride metabolism, we measured expression of key regulatory genes including peroxisome proliferator activated receptor α (PPARα), a master regulator of lipid metabolism which is highly expressed in liver and plays an important role in hepatic lipid metabolism [12]; fatty acid synthase (FASN), which mediates synthesis of fatty acids in the liver [13]; and very long chain acyl-CoA dehydrogenase (VLCAD), which catalyzes the initial stage of lipid β-oxidation [14]. As shown in Figure 4, PPARα mRNA was decreased by almost 50% during HCV infection but was borderline increased in cells expressing HBV. FASN mRNA was increased by both HCV and HBV. Finally, there was a significant decrease in VLCAD mRNA in HCV-infected cells, but not in HBV-expressing cells. Together, these results suggest that during HCV infection although fatty acid synthesis is increased, overall lipid metabolism and fatty acid oxidation is impaired. In contrast, during HBV expression fatty acid oxidation is not impaired and utilized by the cells.

To determine the effects of HCV and HBV on cholesterol synthesis, we examined expression of sterol regulatory element binding protein 2 (SREBP2), a transcription factor which initiates cholesterol synthesis; 3-hydroxy-3-methylglutaryl CoA reductase (HMGCR), a rate-limiting enzyme that mediates cholesterol synthesis in the liver [15]; and low-density lipoprotein receptor (LDLR), which is enriched on the outer surface of hepatocytes and mediates uptake of cholesterol containing low-density lipoprotein (LDL) [16]. In HCV-infected cells there was a significant increase in expression of SREBP2, HMGCR, and LDLR (Figure 4*A*). During HBV expression SREBP2 also increased, but HMGCR and LDLR each decreased by approximately 50% (Figure 4*B*). These results suggest increased intracellular cholesterol in cells infected with HCV but not during HBV expression.

To determine the effect of HBV expression and HCV infection on intracellular lipids, intracellular concentrations of triglyceride, cholesterol, and cholesteryl ester were measured. During HCV infection, intracellular triglyceride (Figure 4*C*) and cholesterol (Figure 4*E*) increased. In contrast, in HBV-expressing cells there was no significant change in intracellular triglyceride (Figure 4*D*) or cholesterol (Figure 4*F*). Although there was a trend towards increased cholesteryl ester with HCV (but not HBV) this was not statistically significant (Supplementary Figure 3). Altogether, our results show that impaired lipid oxidation, increased triglyceride, and cholesterol synthesis and uptake likely contribute to lipid accumulation in cells infected with HCV but not HBV.

### Perturbation of Lactate Metabolism by HBV

In hepatocytes ATP can be produced by either aerobic or anaerobic glycolysis, with variable efficiency. During aerobic glycolysis, glucose is converted to pyruvate and then to acetyl-CoA, which enters the tricarboxylic acid (TCA) cycle, producing 38 ATP molecules per glucose molecule [17]. Anaerobic glycolysis produces only 2 ATP molecules, by converting pyruvate to lactate. Seahorse analysis of live cells (Figure 2) showed that although both HCV and HBV cause mitochondrial dysfunction by inhibiting OXPHOS, only HCV inhibits ATP production by glycolysis.

**Figure 5.**
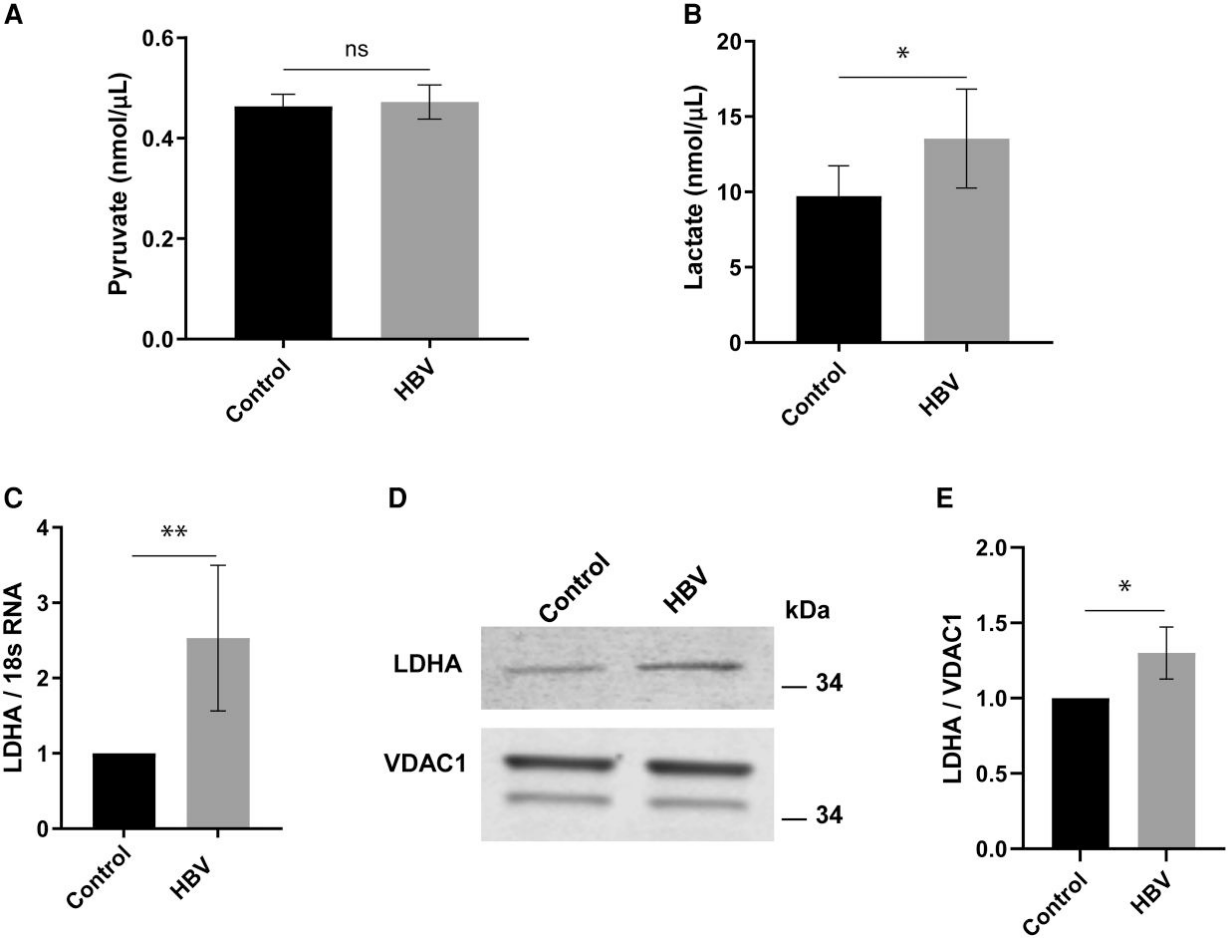
HBV expression promotes lactate accumulation. Effect of HBV expression on cellular pyruvate (*A*) and lactate (*B*) (n = 5). Changes in LDHA mRNA expression (*C*) by real-time PCR. Effect of HBV expression on LDHA protein by western blot (*D*) and densitometry analysis (*E*). Each bar represents an average of n = 3 biological experiments. Error bars represents SEM. *P <0 .05, **P < 0.01. Abbreviations: HBV, hepatitis B virus; LDHA, lactate dehydrogenase A; ns, not significant; PCR, polymerase chain reaction; VDAC1, voltage-dependent anion-selective channel 1.

To look for possible effects of HBV on pyruvate metabolism or anaerobic glycolysis, intracellular concentrations of pyruvate and lactate in HBV-expressing HepAD38 cells and HepG2 controls were quantified. There was no change in pyruvate concentrations in HBV-expressing cells (Figure 5*A*), but there was lactate accumulation (Figure 5*B*), suggesting increased conversion of pyruvate to lactate.

The tetrameric enzyme lactate dehydrogenase (LDH) catalyzes the interconversion of pyruvate to lactate during glycolysis and ensures the availability of sufficient nicotinamide adenine dinucleotide (NAD^+^) to fuel this process [18]. LDHA enhances the conversion of pyruvate to lactate, whereas LDHB favors the reverse reaction [19]. Thus, to further elucidate the mechanism of increased lactate in HBV-expressing HepAD38 cells, LDHA mRNA expression was measured by real-time polymerase chain reaction (PCR). We observed a 2.5-fold increase in LDHA mRNA expression (Figure 5*C*), with increased LDHA protein confirmed by western blot and densitometry (Figure 5*D* and 5*E*).

To determine whether this increase in anaerobic glycolysis is specific to HBV, LDHA mRNA was compared in HCV-infected Huh7 cells and uninfected controls. In contrast to HBV, LDHA expression decreased by 33% during HCV infection (Supplementary Figure 4*A*). This suggests decreased conversion of pyruvate to lactate in HCV-infected cells, consistent with the impaired production of ATP from glycolysis (Figure 2*B*).

### Mitochondrial Proteome Analysis During HBV Expression and HCV Infection

In cells containing HBV, we hypothesized that higher lactate concentrations could perturb metabolic pathways downstream of pyruvate, explaining reduced ATP production. To identify proteins involved, SILAC-based proteomics analysis of mitochondria isolated from HBV-expressing and control cells was performed. For this, HepAD38 and HepG2 cells were cultured for 5 passages in media containing heavy and medium isotopes of arginine and lysine to allow at least 97% incorporation of the labelled amino acid isotopes [20]. Mitochondria from both cell lines were isolated (Supplementary Methods), mixed in equal amounts, and analyzed by LC-MS. The relative intensity of peaks for each mitochondrial protein was used to calculate the SILAC ratio (HepAD38/HepG2). Based on the Mascot score (>90) and the SILAC ratio, 41 mitochondrial proteins were shortlisted from the original data. Supplementary Table 1 shows the list of upregulated and downregulated proteins, while those with no change are listed in Supplementary Table 3.

The altered mitochondrial proteins were submitted to the DAVID 6.8 software package for functional annotation analysis. To perform gene ontology (GO) enrichment, the Functional Annotation Chart available on DAVID was used. GO enrichment with a gene count greater than 3-fold and significant *P* value was preferred. The GO analysis lists all the functional annotation terms, leading to repetition of similar terms comprising the same genes. Therefore, the Functional Annotation Clustering tool available on the DAVID software was used to avoid redundancy and to group similar functional terms. The annotation terms with higher fold change for each cluster of upregulated or downregulated proteins are listed in Supplementary Table 2.

Mass spectrometry revealed upregulation of pyruvate dehydrogenase kinase isoforms PDK1, PDK2, and PDK3 in HBV-expressing cells, with Mascot scores of 296, 107, and 220, respectively (Supplementary Table 1). Consistent with this, DAVID analysis revealed a high fold enrichment in processes associated with pyruvate metabolism (Supplementary Table 2). Annotation cluster 1 for mitochondrial proteins involved in metabolism regulation was upregulated in HBV-expressing cells, with genes inhibiting biosynthesis of acyl-CoA from pyruvate having the highest fold enrichment, particularly PDK.

**Figure 6.**
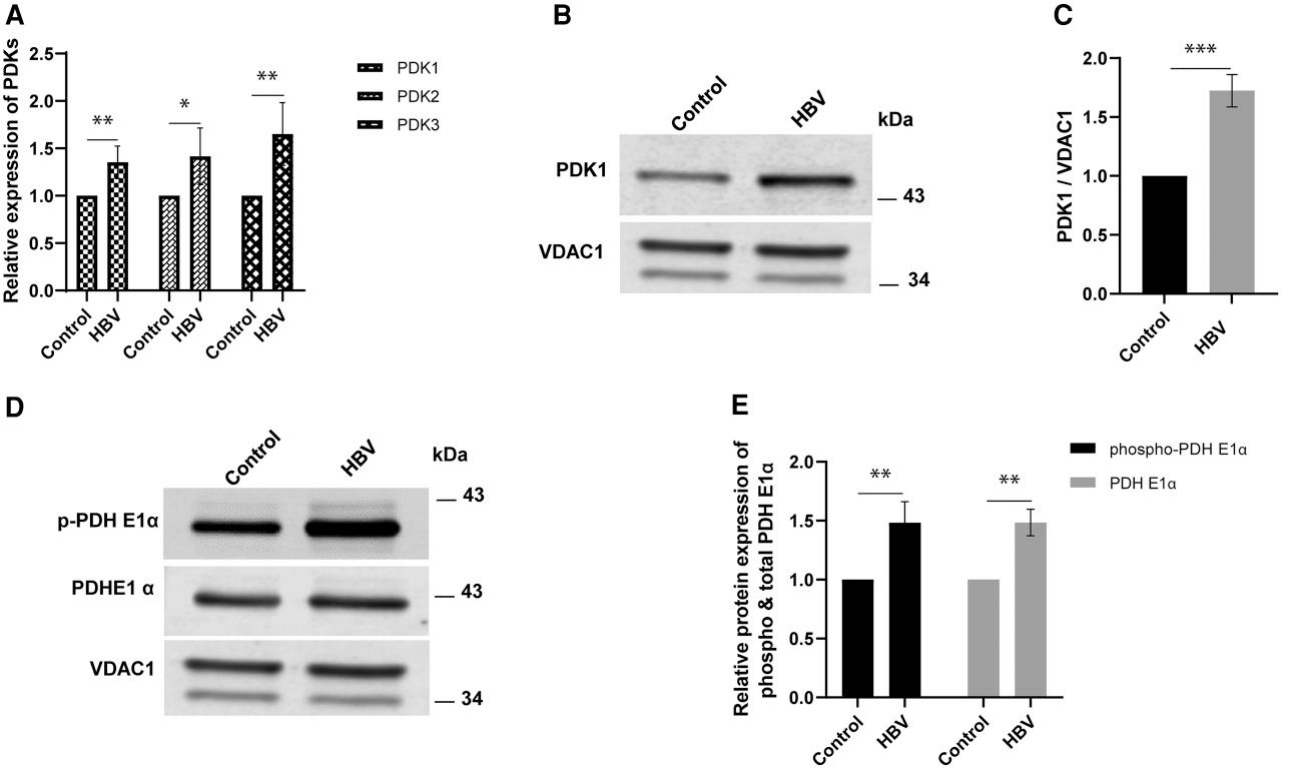
Effect of HBV on pyruvate metabolism. Expression of mRNAs for different PDK isoforms was measured in HBV-expressing cells using real-time PCR (*A*). Effects of HBV expression on PDK1 protein was analyzed by western blot (*B*) and densitometry (*C*). Effects of HBV expression on PDHE1α protein and phosphorylation was analyzed by western blot (*D*) and densitometry (*E*). Each bar represents average of n = 3 biological experiments. Error bars represents SEM. *P < 0.05, **P < 0.01, ***P < 0.001, ****P < 0.0001. Abbreviations: HBV, hepatitis B virus; PDHE1α, pyruvate dehydrogenase subunit E1α; PDK, pyruvate dehydrogenase kinase; p-PDHE1α, phospho-PDHE1α; PCR, quantitative polymerase chain reaction; VDAC1, voltage-dependent anion-selective channel 1.

To confirm this result, mRNA transcript levels for PDK isoforms were measured by real-time PCR. There was a significant increase in expression of PDK1, PDK2, and PDK3 in HBV-expressing cells (Figure 6*A*). Western blot confirmed a 1.7-fold increase of PDK1 protein in mitochondria from HBV-expressing cells (Figure 6*B* and 6*C*).

PDK inhibits acetyl CoA synthesis from pyruvate by regulating pyruvate dehydrogenase complex activity. PDK phosphorylates and inactivates pyruvate dehydrogenase (PDH) subunit E1α, reducing acetyl-CoA availability [21]. Regulation of PDH is essential for inhibiting mitochondrial respiration and production of reactive oxygen species (ROS), as the tricarboxylic acid (TCA) cycle is coupled to the electron transport chain [22].

To determine if increased PDK phosphorylates and thereby inactivates PDH as predicted, concentrations of total and phosphorylated PDH E1α subunit were measured by western blot. A significant increase in phospho-PDH E1α and total PDH E1α was observed in HBV-expressing cells (Figure 6*D* and 6*E*). These data suggest that the HBV-induced increase in PDKs inactivates PDH, inhibiting conversion of pyruvate to acetyl-CoA, resulting in reduced ATP production through mitochondrial oxidative phosphorylation.

To confirm specificity for HBV, the experiment was repeated for HCV-infected cells. Expression of PDK1, PDK2, and PDK3 was significantly reduced to 50% or less during HCV infection (Supplementary Figure 4*B*). This suggests that in HCV-infected cells, decreased PDK would not inactivate the PDH complex, allowing conversion of pyruvate to acetyl-CoA for subsequent entry into the TCA cycle.

To complement our HBV proteomics analysis, a similar SILAC analysis was performed on mitochondria isolated from HCV-infected and -uninfected Huh7. The list of mitochondrial proteins differentially regulated during HCV infection is provided in Supplementary Table 4. There was a 50% decrease in pyruvate carboxylase (PC) expression in HCV-infected cells compared to controls (ie, PC was 2-fold higher in uninfected than uninfected cells). Consistent with our results, reduced PC has been reported in cells expressing HCV NS5A, which increased acetyl-CoA to facilitate lipid accumulation [23]. In our analysis, the ATP synthase β subunit was significantly reduced in HCV-infected cells (*P* = .04) and there was a trend towards reduced expression of ATP synthase subunit α (*P* = .09). Located on the inner mitochondrial membrane, ATP synthase catalyzes the formation of ATP [11]. Thus reduced ATP synthase in HCV-infected cells is consistent with our Seahorse results showing decreased ATP-linked respiration (Figure 1). Finally, we observed decreased expression of trifunctional protein subunit α (*P*=0.02), consistent with a report that showed downregulation of mitochondrial trifunctional protein attenuates lipid oxidation during HCV infection [9], and with the reduced VLCAD expression and increased lipid accumulation we observed with HCV infection.

**Figure 7.**
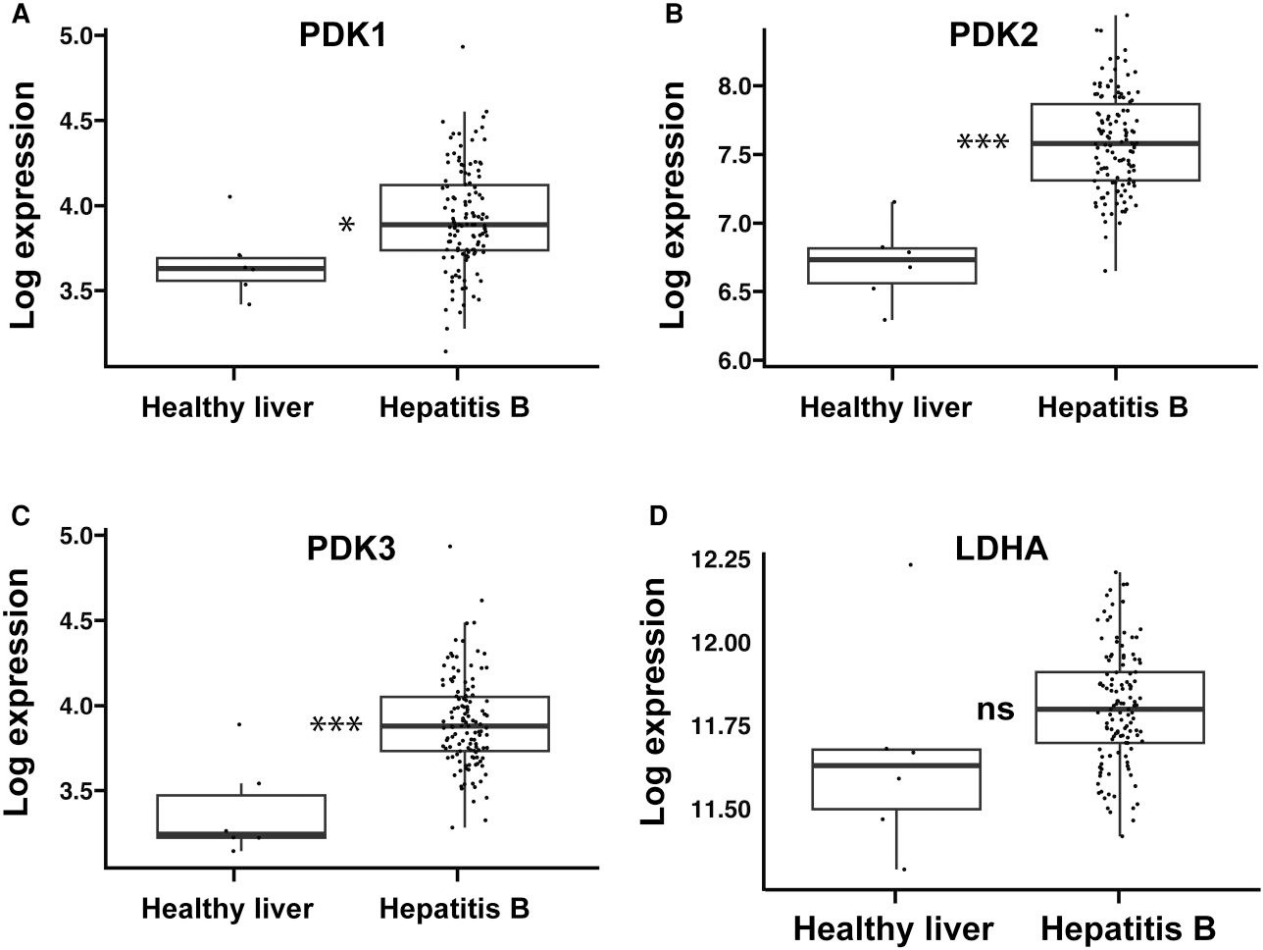
Gene expression analysis of pyruvate metabolism in patients with chronic HBV. Expression of key genes involved in pyruvate metabolism in liver biopsies from people with chronic hepatitis B and healthy controls (GSE83148) [24]. Expression of PDK1–3 (*A*–*C*) and LDHA (*D*). *P <0 .05, ***P < 0.001. Abbreviations: HBV, hepatitis B virus; LDHA, lactate dehydrogenase A; ns, not significant; PDK, pyruvate dehydrogenase kinase.

To assess the clinical impact of our findings, we analyzed the effects of HBV infection on gene expression in human liver (GSE83148) [24]. Consistent with our in vitro findings, in people with chronic hepatitis B there was significantly increased expression of PDK1, PDK2, and PDK3 compared to healthy liver (Figure 7*A*–*C*), with a strong trend towards increased expression of LDHA (*P* =0.057; Figure 7*D*). Similar analysis of online datasets for people with hepatitis C found no consistent changes in expression of lipid-associated genes, likely due to study heterogeneity. However, liver biopsy studies have shown altered expression of genes involved in lipogenesis and lipolysis that promote steatosis [10], consistent with the lipid accumulation we and others have observed with HCV infection.

## DISCUSSION

HCV (48%) and HBV (47%) account for most of the 1.4 million deaths each year caused by viral hepatitis, either from acute infection, cirrhosis, or liver cancer [1]. Typical clinical features of chronic hepatitis C include liver steatosis, insulin resistance, and diabetes mellitus, due to impaired lipid and glucose metabolism [2, 25–27], features that are not associated with hepatitis B [4, 11]. The reasons for these contrasting effects on liver metabolism are not well understood, so we compared the effects of each virus on liver metabolism and mitochondrial function. As summarized in our Graphical Abstract, our results suggest that both HCV and HBV inhibit mitochondrial ATP production, with HCV also inhibiting glycolysis.

Enriched with mitochondria, liver is the principal site for metabolic activities [28]. Mitochondria generate the energy rich ATP molecules required to fuel cellular activities [29], so hepatitis viruses depend on mitochondria for their replication and virus production [30]. However, mitochondria are also involved in the innate antiviral immune response [31], presenting a potential conflict for viruses that replicate in the liver. Mitochondrial injury and oxidative stress have been reported previously for both HCV and HBV infections [32, 33], with various potential mechanisms suggested. Most HCV proteins associate with and affect the function of mitochondria [34]. The HBx protein of HBV associates with mitochondria causing oxidative stress [35].

Our analysis of mitochondria in live cells provides novel insights into the mechanisms of HBV- and HCV-induced dysfunction. We confirmed that reduced ATP production was due to a reduction in mitochondrial function and not due to a reduction in mitochondria number, using a validated method with 2 separate markers [9]: PGC-1α, a major regulator of mitochondrial biogenesis [36]; and mitochondria DNA copy number, which is proportional to the number of mitochondria [37]. Reduced mitochondrial function [9] and lower levels of cytoplasmic ATP [38] have been observed in HCV-infected cells, but to our knowledge this has not been reported previously for HBV. We also demonstrated decreased mitochondrial membrane potential for both HCV and HBV, consistent with previous reports [39, 40].

We observed impaired lipid oxidation in HCV-infected cells, reducing the availability of acetyl-CoA to feed into the TCA cycle and contributing to reduced mitochondrial ATP production. Reduced lipid oxidation leads to intracellular lipid accumulation, consistent with the 2.5-fold risk of steatosis (fatty liver) in people with chronic hepatitis C [9, 41]. In people with hepatitis B, steatosis is likely reduced [5, 42]. Consistently, we did not see any impact of HBV on lipid oxidation or lipid accumulation.

In people with hepatitis B, our novel results suggest that mitochondrial dysfunction is due to HBV-induced changes in pyruvate metabolism. We observed induction of LDHA, which increases conversion of pyruvate to lactate, consistent with a recent report that excess lactate during HBV infection suppresses innate cellular immunity [43]. Our finding that HBV induces expression of the PDK family of proteins provides further mechanistic insight, as PDK inhibits the conversion of pyruvate to acetyl-CoA by phosphorylating and inactivating PDH [21]. Reduced availability of acetyl-CoA decreases ATP production via mitochondrial OXPHOS, as we observed, and increases the availability of pyruvate for lactate conversion. The subsequent accumulation of lactate causes oxidative stress, likely contributing to the much higher rates of hepatocellular carcinoma observed in people with chronic hepatitis B than hepatitis C [6].

In summary, our data show mitochondrial dysfunction in HCV infection and HBV expression, but the mechanisms differ. For HCV, impaired glycolysis and lipid oxidation reduces ATP production through oxidative phosphorylation and causes lipid accumulation and steatosis. In contrast, HBV-induced mitochondrial dysfunction is independent of lipid metabolism and is caused by disturbed pyruvate metabolism. HBV inhibits pyruvate to acetyl-CoA conversion by inducing PDK. This stimulates increased conversion of pyruvate to lactate via upregulated LDHA, resulting in lactate accumulation, oxidative stress, and mitochondrial toxicity.

Our results provide new insights into the different complications of chronic hepatitis C and hepatitis B, that is, steatosis and liver cancer respectively. Increased oxidative stress with HBV infection might account for the rapid disease progression compared to HCV and could contribute to liver cancer development. For people with hepatitis B, targeting LDHA or PDK could offer new strategies to reduce oxidative stress and thus the risk of liver cancer.

## Supplementary Data

Supplementary materials are available at *The Journal of Infectious Diseases* online (http://jid.oxfordjournals.org/). Supplementary materials consist of data provided by the author that are published to benefit the reader. The posted materials are not copyedited. The contents of all supplementary data are the sole responsibility of the authors. Questions or messages regarding errors should be addressed to the author.

## Supplementary Material

jiae210_Supplementary_Data

## Notes

***Author contributions*.** S. P. S., A. K., E. S. E. T., and M. W. D. conceived and designed the study. S. P. S., M. G., and H. A. performed experiments and data analysis. E. D. performed mass spectrometry experiments. B. G. performed GEO dataset analysis. S. P. S., H. A., and M. W. D. prepared the manuscript. S. P. S., A. K., J. G., T. T., and M. W. D. reviewed and edited the manuscript. All authors have read and agreed to the final version of the manuscript.

***Financial support*.** This work was supported by the National Health and Medical Research Council, Australia (project grant number 1047417 and program grant number 1053206); and the Robert W. Storr bequest to the Sydney Medical Foundation, University of Sydney. S. P. S. was supported by a University of Sydney Research Training Program Stipend Scholarship; and the Westmead Institute for Medical Research Top-up Grant. A. K. was supported by a University of Sydney Postdoctoral Research Fellowship.
