# Supplementary material for: Hepatitis B Virus and Hepatitis C Virus Affect Mitochondrial Function Through Different Metabolic Pathways, Explaining Virus-Specific Clinical Features of Chronic Hepatitis
Source: J Infect Dis. 2024 Apr 24;230(5):e1012–22. doi: 10.1093/infdis/jiae210 (PMC11566039; doi:10.1093/infdis/jiae210)
Supplement: jiae210_Supplementary_Data [file jiae210_supplementary_data.docx]

## 1. SUPPLEMENTARY FIGURES


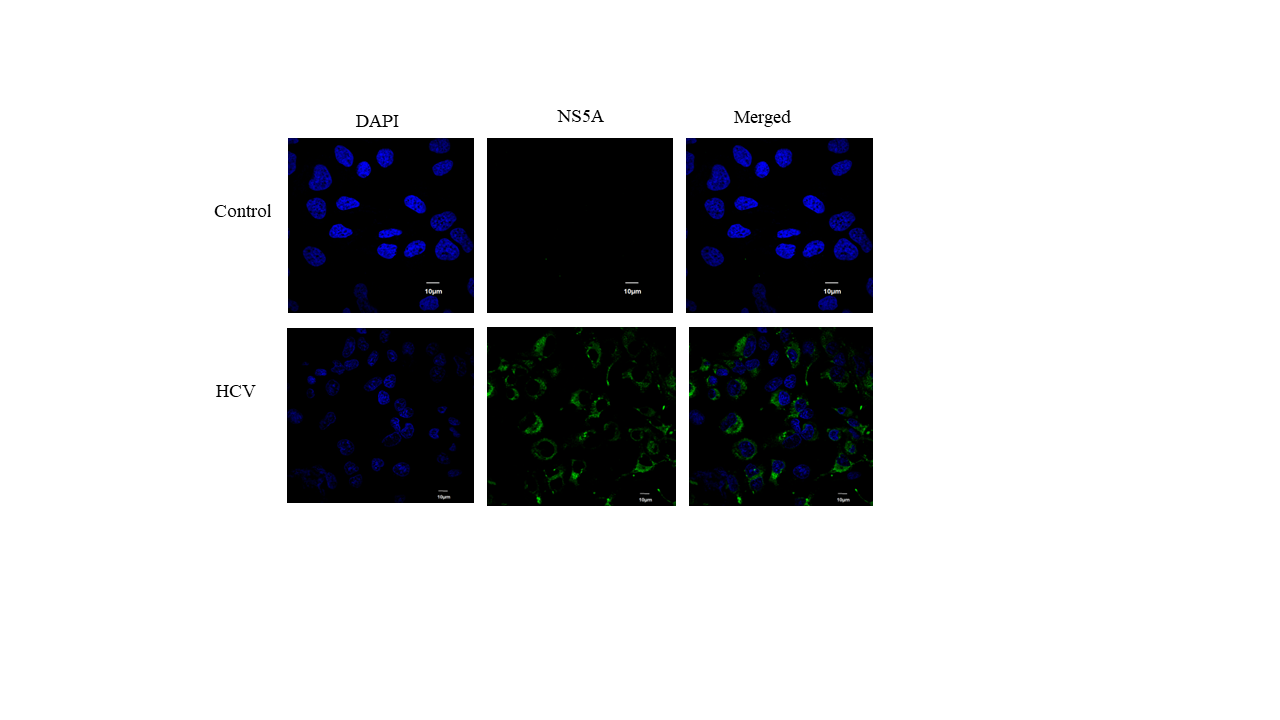


Supplementary Figure 1. HCV NS5A protein expression in HCV(JFH1) infected cells

Uninfected Huh7 and HCV (JFH1) transfected cells were labelled with antibody against the HCV protein NS5A. The presence of fluorescence (green) surrounding the DAPI stained nuclei (blue) indicates the expression of HCV NS5A. The scale bar represents 10 µm.


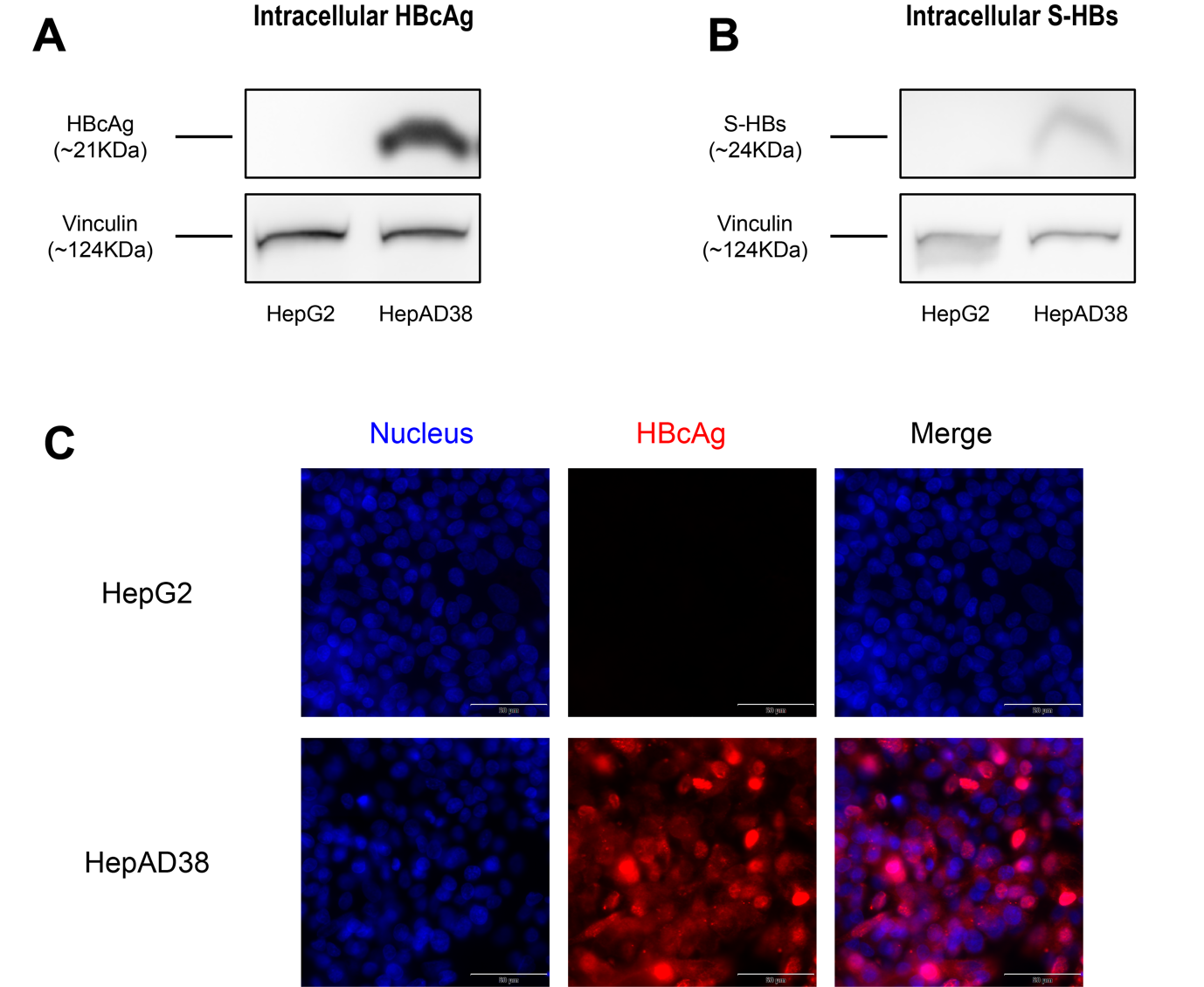


Supplementary Figure 2. HBV protein expression in HepAD38 cells

HepAD38 cells and HepG2 controls were cultured in the absence of tetracycline.
**(A)** Intracellular hepatitis B core antigen (HBcAg) expression was analysed by Western blot. Vinculin was used as loading control. **(B)** Intracellular small hepatitis B s antigen (S‑HBs) expression was analysed by Western blot. Vinculin was used as loading control. **(C)** HBcAg (red) expression was analysed by immunofluorescence. Nuclei are stained in blue using DAPI (scale bar: 50 μm). (n=3)


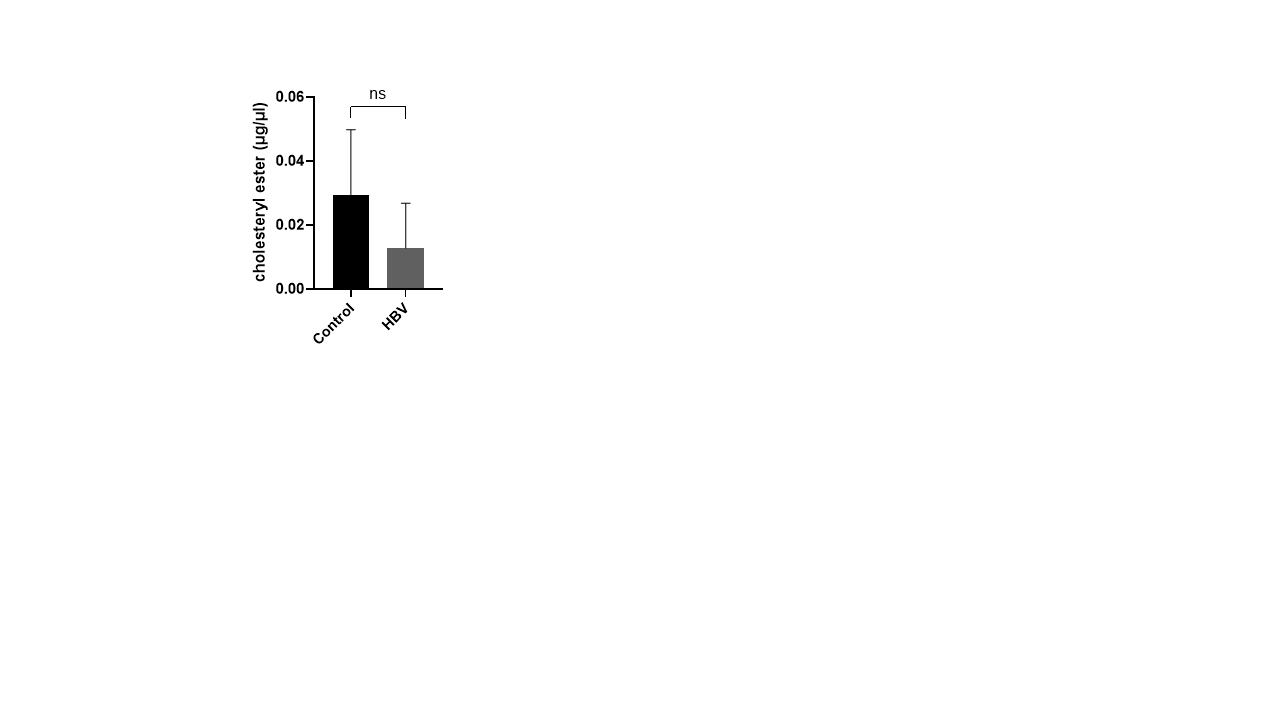

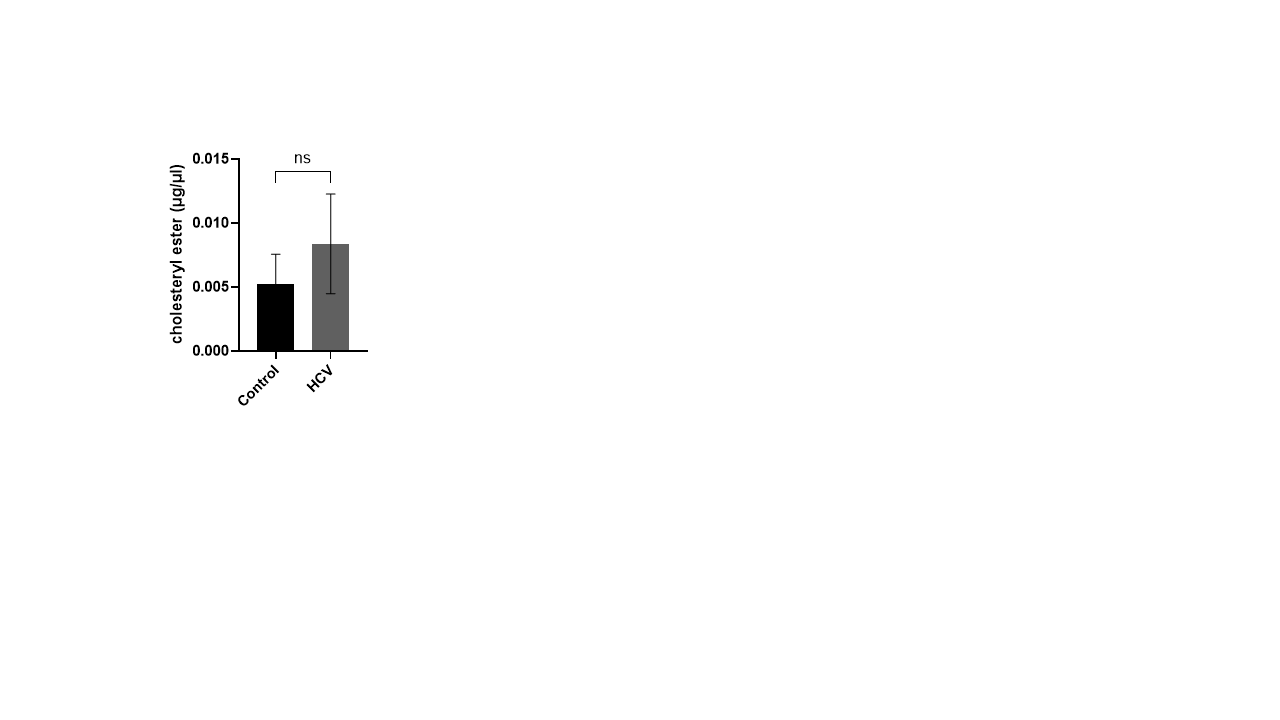
 **A B**

**Supplementary Figure 3. Effect of HCV infection on cholesteryl ester**

Effects of (A) HCV and (B) HBV on cellular cholesteryl ester . Each bar represents the mean of three biological experiments. Error bars represent SEM. ns - not significant.

**A B**


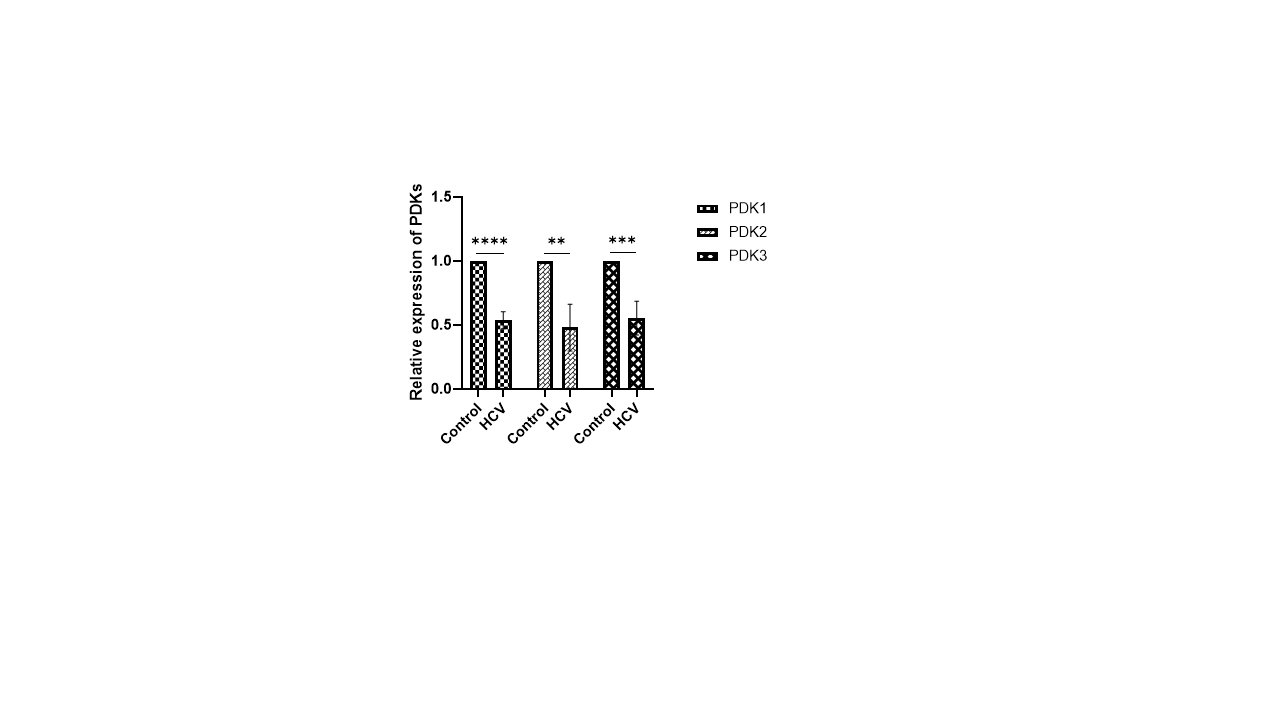

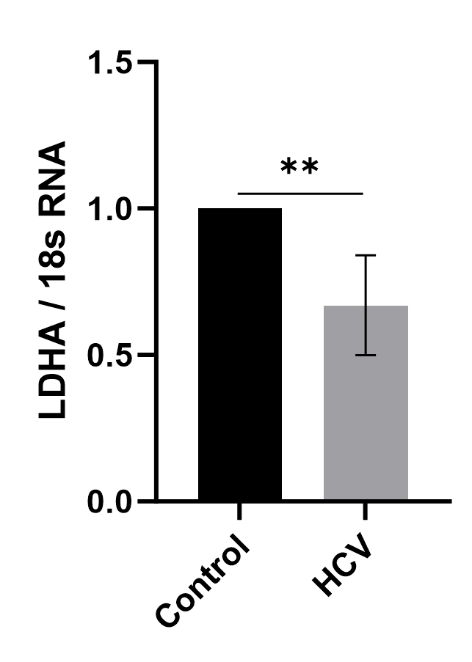


**Supplementary Figure 4. Effect of HCV infection on expression of LDHA and PDK**

To confirm that the perturbation of pyruvate metabolism is specific to HBV, mRNA levels for **(A)** LDHA and **(B)** PDKs were measured in control and HCV infected cells by real time PCR.
** p<0.01, *** p<0.001, **** p<0.0001

## 2. SUPPLEMENTARY TABLES

| **No** | **Gene Symbol** | **Name** | **SILAC Ratio (HepAD38/HepG2)** | **Mascot score** | **# peptides** |
| --- | --- | --- | --- | --- | --- |
| **Upregulated in HepAD38 cells (HBV)** | | | | |  |
| 1 | SDHAF2 | Succinate dehydrogenase assembly factor 2 | 5.75 | 304 | 8 |
| 2 | PDK3 | Pyruvate dehydrogenase (acetyl-transferring) kinase isozyme 3 | 5.55 | 220 | 8 |
| 3 | FPGS | Folylpolyglutamate synthase, mitochondrial | 3.44 | 155 | 8 |
| 4 | ALAS1 | 5-aminolevulinate synthase, nonspecific, mitochondrial | 3.16 | 152 | 7 |
| 5 | OMA1 | Metalloendopeptidase OMA1 | 2.99 | 97 | 7 |
| 6 | PDK1 | Pyruvate dehydrogenase (acetyl-transferring) kinase isozyme 1 | 2.93 | 296 | 13 |
| 7 | OGDHL | 2-oxoglutarate dehydrogenase-like | 2.84 | 1209 | 21 |
| 8 | COQ8B | Atypical kinase | 2.32 | 220 | 5 |
| 9 | LDHA | L-lactate dehydrogenase A | 2.27 | 404 | 11 |
| 10 | PDK2 | Pyruvate dehydrogenase (acetyl-transferring) kinase isozyme 2 | 2.16 | 107 | 5 |
| 11 | ALKBH7 | Alpha-ketoglutarate-dependent dioxygenase alkB homolog 7 | 2.11 | 91 | 4 |
| 12 | MRM2 | rRNA methyltransferase 2 | 2.08 | 110 | 6 |
| 13 | BCKDK | 3-methyl-2-oxobutanoate dehydrogenase kinase | 2.01 | 222 | 7 |
| **Downregulated in HepAD38 cells** | | | | | |
| 1 | BDH1 | D-beta-hydroxybutyrate dehydrogenase | 0.46 | 234 | 9 |
| 2 | OXCT1 | Succinyl-CoA:3-ketoacid coenzyme A transferase 1 | 0.2 | 258 | 10 |
| 3 | ACSM2B | Acyl-coenzyme A synthetase | 0.07 | 275 | 15 |

**Supplementary Table 1.** **Changes in mitochondrial proteins during HBV expression, analysed using Stable Isotope Labelling by Amino acids in Cell Culture (SILAC)**

HBV expressing HepAD38 cells and HepG2 controls were cultured with isotope-labelled amino acids, mitochondria were purified and analysed by mass spectrometry. Mitochondrial proteins that increased or decreased at least two-fold are listed*.*

| **Category** | **Term** | **Count** | **Genes** | **Fold enrichment** | **Benjamini p value** |
| --- | --- | --- | --- | --- | --- |
| **Upregulated in HepAD38 cells** | | | | | |
| **Annotation Cluster 1 Enrichment Score: 2.77** | | | | | |
| GOTERM_BP_FAT | GO:0010510 Regulation of acyl-CoA biosynthetic process from pyruvate | 3 | PDK1, PDK2, PDK3 | 320 | 1.3E-3 |
| GOTERM_BP_FAT | GO:0042762  Regulation of sulphur metabolic process | 3 | PDK1, PDK2, PDK3 | 190 | 2.8E-3 |
| GOTERM_BP_FAT | GO:0051193  Regulation of cofactor metabolic process | 3 | PDK1, PDK2, PDK3 | 72 | 1.3E-2 |
| GOTERM_MF_FAT | GO:0004672  Protein kinase activity | 5 | BCKDK, COQ8B, PDK1, PDK2, PDK3 | 9.9 | 2E-2 |
| GOTERM_MF_FAT | GO:0016773  Phosphotransferase activity, alcohol group as acceptor | 5 | BCKDK, COQ8B, PDK1, PDK2, PDK3 | 8.2 | 3E-2 |
| **Annotation Cluster 2 Enrichment Score: 2.66** | | | | | |
| GOTERM_BP_FAT | GO:0032787  Monocarboxylic acid metabolic process | 6 | ALKBH7, LDHA, OGDHL, PDK1, PDK2, PDK3 | 14 | 1.3E-3 |
| GOTERM_BP_FAT | GO:0044723  Single-organism carbohydrate metabolic process | 6 | OMA1, LDHA, OGDHL, PDK1, PDK2, PDK3 | 12 | 2.0E-3 |
| GOTERM_BP_FAT | GO:0005975  Carbohydrate metabolic process | 6 | PDK1, PDK2, PDK3, LDHA, OMA1, OGDHL | 9.9 | 3.8E-3 |
| **Downregulated in HepAD38 cells** | | | | | |
| **Annotation Cluster 1 Enrichment Score: 3.02** | | | | | |
| GOTERM_CC_FAT | GO:0005759  Mitochondrial matrix | 3 | BDH1, OXCT1, ACSM2B | 3.4E1 | 1.2E-2 |
| GOTERM_CC_FAT | GO:0044429  Mitochondrial part | 3 | BDH1, OXCT1, ACSM2B | 1.5E1 | 3.2E-2 |

Supplementary Table 2. Functional annotation clustering using DAVID 6.8

| **No** | **Gene Symbol** | **Name** | **SILAC Ratio (HepAD38/HepG2)** | **Mascot score** | **# peptides** |
| --- | --- | --- | --- | --- | --- |
| **No change in HepAD38 cells (HBV)** | | | | |  |
| 1 | ABCB10 | ATP-binding cassette sub-family B member 10 | 1.57 | 168 | 6 |
| 2 | PDHA1 | Pyruvate dehydrogenase E1 component subunit alpha | 1.41 | 3446 | 24 |
| 3 | DMAC2L | ATP synthase subunit s | 1.4 | 278 | 8 |
| 4 | ACAD9 | Acyl-CoA dehydrogenase family member 9 | 1.39 | 1757 | 25 |
| 5 | PDHB | Pyruvate dehydrogenase E1 component subunit beta | 1.37 | 2925 | 15 |
| 6 | ATP5MD | ATP synthase membrane subunit DAPIT | 1.37 | 140 | 3 |
| 7 | ACADVL | Very long-chain specific acyl-CoA dehydrogenase | 1.35 | 6008 | 33 |
| 8 | ATP5F1C | ATP synthase subunit gamma | 1.34 | 3349 | 17 |
| 9 | ATP5PD | ATP synthase subunit d | 1.31 | 1341 | 15 |
| 10 | ACADM | Medium-chain specific acyl-CoA dehydrogenase | 1.26 | 2226 | 24 |
| 11 | ATP5MG | ATP synthase subunit g | 1.22 | 308 | 5 |
| 12 | CPT2 | Carnitine O-palmitoyl transferase 2 | 1.21 | 1428 | 25 |
| 13 | ATP5F1A | ATP synthase subunit alpha | 1.2 | 27030 | 43 |
| 14 | ATP5PB | ATP synthase F (0) subunit B1 | 1.19 | 2578 | 19 |
| 15 | ATP5PO | ATP synthase subunit O | 1.19 | 2761 | 14 |
| 16 | ATP5F1D | ATP synthase subunit delta | 1.18 | 822 | 4 |
| 17 | ATP5F1B | ATP synthase subunit beta | 1.17 | 30928 | 31 |
| 18 | TFAM | Transcription factor A | 1.15 | 205 | 5 |
| 19 | ETFDH | Electron transfer flavoprotein – ubiquinone oxidoreductase | 0.97 | 484 | 14 |
| 20 | CS | Citrate synthase | 0.9 | 7055 | 26 |
| 21 | SOD2 | Superoxide dismutase | 0.9 | 3180 | 12 |
| 22 | HADHA | Trifunctional enzyme subunit alpha | 0.89 | 15467 | 45 |
| 23 | HMGCL | Hydroxymethylglutaryl-CoA lyase | 0.87 | 732 | 10 |
| 24 | HADHB | Trifunctional enzyme subunit beta | 0.86 | 9762 | 31 |
| 25 | PC | Pyruvate carboxylase | 0.56 | 10394 | 60 |

Supplementary Table 3. Mitochondrial proteins with no change during HBV expression

| **Name** | **SILAC ratio (Huh7/JFH1)** | **Peptides (95%)** | **Sequence coverage (%)** | **P value** | **Error factor** |
| --- | --- | --- | --- | --- | --- |
| NADH dehydrogenase [ubiquinone] iron-sulfur protein 6 | 0.02 | 2 | 60.48 | 0.0001 | 1.45 |
| Pyruvate carboxylase | 2.01 | 78 | 73.6 | 0.01 | 1.70 |
| Succinate dehydrogenase cytochrome b560 subunit | 2.64 | 2 | 51.48 | 0.008 | 1.43 |
| Leucine-rich PPR motif-containing protein | 2.0 | 110 | 74.53 | 0.02 | 1.78 |
| Trifunctional enzyme subunit alpha | 1.91 | 75 | 72.21 | 0.02 | 1.73 |
| NADH-ubiquinone oxidoreductase 75 kDa subunit | 2.36 | 35 | 68.23 | 0.03 | 2.19 |
| Enoyl-CoA hydratase domain-containing protein 3 | 2.18 | 3 | 39.27 | 0.03 | 1.98 |
| ATP synthase subunit beta | 2.36 | 91 | 90.17 | 0.04 | 2.22 |
| Nucleoside diphosphate kinase | 2.28 | 2 | 74.33 | 0.04 | 2.0 |
| ATP synthase subunit alpha | 1.92 | 54 | 69.08 | 0.09 | 2.18 |
| Aldehyde dehydrogenase, mitochondrial | 2.19 | 33 | 61.7 | 0.05 | 2.21 |
| 60 kDa heat shock protein | 2.25 | 133 | 90.4 | 0.05 | 2.29 |

Supplementary Table 4. Mitochondrial proteome analysis during HCV infection

The table lists abundance of mitochondrial proteins during HCV infection. Control Huh7 and HCV JFH1 infected Huh7 were cultured with isotopes of amino acids, mitochondria isolated and analysed by mass spectrometry. For each protein, the table summarises the ratio of protein abundance in HCV JFH1 infected Huh7 cells compared to control Huh7, the number of peptides used for protein identification, and relative sequence coverage. Protein ratios with a p value less than 0.05 and error factor less than 2 were considered statistically significant.

| **Gene** | **Primer Sequence** |
| --- | --- |
| PPARα | FP - GGCAAGACAAGCTCAGAAC  RP - TTATCTATGAAGCAGGAAGCAC |
| PGC-1α | FP - TGCCCTGGATTGTTGACATGA  RP - TTTGTCAGGCTGGGGGTAGG |
| LDLR | FP - GGCAGTGTGACCGGGAATATG  RP - TTCGCCGCTGTGACACTTG |
| HMGCR | FP - GCCTGGCTCGAAACATCTGAA  RP- TGACCTGGACTGGAAACGGATA |
| SREBP2 | FP - CAAGGCCCTGGAAGTGACA  RP - AGGAACTCTGCTGCCCATCTG |
| VLCAD | FP - AAAATTCACAACTTTGGGCTGA  RP - CCCTGGTCCATGTTAGCACT |
| 18s RNA | FP - CAGCCACCCGAGATTGAGCA  RP - TAGTAGCGACGGGCGGTGTG |
| FASN | FP - ACAGGGACAACCTGGAGTTCT  RP - CTGTGGTCCCACTTGATGAGT |
| LDHA | FP - TTGGTCCAGCGTAACGTGAAC  RP - CCAGGATGTGTAGCCTTTGAG |
| PDK1 | FP - CGGATCAGAAACCGACACA  RP - ACTGAACATTCTGGCTGGTGA |
| PDK2 | FP - AAGGACACCTACGGCGATG  RP - ATGGAGATGCGGCTGAGG |
| PDK3 | FP - TTAATAAGTCCGCATGGCGC  RP - TGAAGCATCCCTGGGTTCAC |

Supplementary Table 5. List of PCR primers with their sequences

| **Antibodies** | **Supplier** | **Catalogue No** |
| --- | --- | --- |
| Anti-VDAC1 | Abcam | ab14734 |
| Anti-PDK1 | Abcam | ab207450 |
| Anti-Pyruvate dehydrogenase E1-alpha subunit (phospho S293) | Abcam | ab177461 |
| Anti-Pyruvate dehydrogenase E1-alpha subunit | Abcam | ab110330 |
| Anti-Lactate dehydrogenase | Abcam | ab52488 |
| IRDye 800CW anti-mouse | Licor | LCR-926-32210 |
| IRDye 800CW anti-rabbit IgG | Licor | LCR-926-32211 |
| Anti-HBsAg | Novus Biologicals | NB100-62652 |
| Anti-HBcAg | Tokyo Future Style | 2AHC24 |
| Anti-Vinculin | Abcam | Ab129002 |

Supplementary Table 6. List of primary and secondary antibodies

**3. SUPPLEMENTARY METHODS**

### Cell culture

The human hepatoma cell line Huh7 and human hepatoblastoma cell line HepG2 were a kind gift from Professor John McLauchlan (University of Glasgow, UK). Huh7 and HepG2 cells were grown in Dulbecco’s Modified Eagle Medium (DMEM) (Lonza, # 12-604F) containing L-Glutamine and 4.5g/L D-Glucose supplemented with 10% (w/v) foetal bovine serum (FBS) (Gibco, # 3160602).

HepAD38, a derivative of the HepG2 cell line which stably expresses the HBV genome was a kind gift from Professor Stephen Locarnini (Doherty Institute, Melbourne). These cells were cultured in Dulbecco’s Modified Eagle Medium: Nutrient mixture F12 (DMEM/F12) supplemented with 10% (w/v) foetal bovine serum, 50 mg/ml Penicillin Streptomycin, 50 mg Kanamycin and 50 mg Geneticin. Cells were incubated at 37 ˚C with a constant supply of 5 % CO_2_.

### HCV RNA transcription and electroporation

The HCV genotype 2a plasmid, JFH1 (Japanese Fulminant Hepatitis), was a kind gift from Professor Takaji Wakita (National Institute of Infectious Disease, Tokyo, Japan). HCV RNA was prepared according to a published protocol [1] using T7 RiboMAX Express RNAi System (Promega). 2 μl of HCV (JFH1) RNA was mixed with 1.5 million Huh7 cells to perform electroporation in a 4 mm cuvette with a single pulse at 340 V and 975 μF. The electroporated cells were transferred to a T25 flask and incubated at 37 °C in a CO2 incubator until confluent (around 5-7 days).

### Seahorse mitochondrial assays

### Uninfected and HCV (JFH1) infected Huh7 cells were plated at a density of 8 $\boldsymbol{\times}$10^4^ cells/well. HepG2 and HepAD38 cells were plated at a density of 10^5^ cells/well. The assays were performed as per the manufacturer’s protocol.

### Infected and uninfected cells were cultured overnight in Seahorse tissue culture microplates. Optimal concentrations of oligomycin, FCCP and rotenone/antimycin A were added to the hydrated cartridge and analysed using an Agilent Seahorse XF Analyser.

### For Huh7/HCV Seahorse XF Mito Stress test (# SEA103015100), 1 µM oligomycin, 0.25 µM FCCP (carbonyl cyanide-p-trifluoromethoxyphenylhydrazone) and 0.5 µM rotenone/antimycin A were used. For HepG2/HepAD38, 2 µM oligomycin, 0.5 µM FCCP and 0.5 µM rotenone/antimycin A were used respectively.

### For the XF Real-time ATP rate assay (# SEA103592100) a final concentration of 1.5 µM and 0.5 µM of oligomycin and rotenone/antimycin A were used for Huh7 cells, whereas 2 µM oligomycin and 0.5 µM rotenone/antimycin A were used for HepG2. The Oxygen Consumption Rate (OCR) was normalised with the protein concentration.

### Immunofluorescence for hepatitis C

Uninfected and HCV (JFH1) infected Huh7 were plated on a cover slip placed in a 24 well tissue culture plate until 70% confluent. The media was removed, the cells were washed with PBS and fixed with 4% paraformaldehyde (PFA) for 10 min at room temperature. Cells were permeabilised and blocked using 1 $\times$ PBS containing 5% serum and 0.3% Triton X-100. The cells were washed thrice with PBS. Incubation with primary antibody was carried out in 1 $\times$ PBS containing 1% BSA (Bovine Serum Albumin) and 0.35% Triton X-100 at 4 °C overnight. The next day, cells were washed thrice with PBS, incubated with secondary antibody for 1 h at room temperature followed by three washes with PBS. The cover slips were mounted with ProLong Gold antifade reagent with DAPI (4’,6-diamidino-2-phenylindole) (Thermo Fisher Scientific, catalogue # P36941) and imaged in Olympus FV 1000 confocal laser microscope using a 40$\times$ oil objective.

### Immunofluorescence for hepatitis B

Cells were fixed for immunofluorescence with 4% PFA at room temperature for 30 min. Cells were then washed with three times with PBS for 5 min on a rocker. Cells were permeabilised with 0.25% Triton X-100/PBS and incubated at room temperature for 30 min followed by three times wash with PBS for 5 min on a rocker. Primary antibody (1:1000 dilution of Anti-HBcAg rabbit polyclonal antibody (Cell Marque, Cat# 216A) diluted in 2% BSA/PBS was added to wells to incubate at 4°C overnight on a rocker. On the next day, cells were washed four times with PBS for 5 min on a rocker. Anti-rabbit Polyclonal IgG conjugated AF546 (Invitrogen, Cat #A11010) was 1:1000 diluted in 2% BSA/PBS with 1:1000 Hoechst33342 was added to the cells for 1 h at room temperature on a rocker covered with foil. Cells were washed four times with PBS for 5 min on a rocker. Coverslips were fixed onto glass slides using ProLong Gold Antifade mountant and imaged using an Olympus VS120 Virtual Slide Scanner. Image processing was performed using Olympus Olyvia software.

### Western blotting

Cellular proteins were extracted using radio immunoprecipitation assay buffer (RIPA) and protein in whole-cell or supernatant lysates was quantified by the DC Protein Assay (Bio-rad, Cat #5000111) according to manufacturer’s instructions. For SDS-PAGE, (10-12% resolving and 5% stacking gel) equal amounts of protein were mixed with 5 $\times$loading buffer and heated at 100 °C for 5 min. The protein samples were loaded onto the gel and electrophoresed at 80V for 1.5 to 2 h in 1 $\times$ running buffer and transferred to a polyvinylidene fluoride (PVDF) membrane at 100 V for 2 h at 4 ˚C.

The membrane was blocked in 5% bovine serum albumin (BSA) and incubated overnight with primary antibody in 5% BSA. The next day, the membranes were washed thrice with 1 $\times$ TBST (Tris-Buffered Saline, 0.1% Tween 20) and incubated with corresponding secondary antibody for 1 h at room temperature, followed by three washes with 1 $\times$ TBST and imaged using a LICOR Odyssey imager. The intensity of the bands was quantified using ImageJ software v1.52a.

For HBV protein western blots, 1:1000 dilution was used for the primary antibodies in 5% skim milk. Protein bands were visualised using 1:10 or 1:5 mixture of SuperSignal West Femto Maximum Sensitivity Substrate (Thermo Fisher Scientific, Cat #34094) and SuperSignal West Pico PLUS Chemiluminescent Substrate (Thermo Fisher Scientific, Cat #34580) on ChemiDoc Touch Imaging System (Bio-Rad).

### RNA and DNA isolation

RNA and DNA isolation from control and infected cells were preformed using FavorPrep Tissue Total RNA mini kit (Favorgen ,# FATRK001-2) and QIAamp DNA mini kit (Qiagen, # 51304) respectively as per the manufacturer’s protocol. RNA was stored at -80 ˚C or used for cDNA synthesis and DNA at -20 ˚C respectively after quantification using the NanoDrop ND 100 spectrophotometer.

### Reverse transcription and qPCR

Reverse transcription of cDNA was performed using MMLV Reverse Transcriptase (Promega) or qScript cDNA SuperMix (Gene Target Solutions) according to the manufacturer’s protocol and stored at -20 ˚C for further use.

Quantitative real-time PCR was performed on a BioRad 96 or 384 well plate instrument. Equal amounts of cDNA were mixed with SYBR Green PCR master mix, 0.2 µM gene specific forward and reverse primers, and made up to 15 µl with RNase-free water. Relative gene expression was normalised with 18s RNA and compared with control. The fold change was calculated by double delta C_t_ method.

To calculate mitochondrial DNA (mtDNA) copy number, a human mtDNA monitoring primer set (Takara Bio USA ,# 7246) was used, consisting of primer sets to detect mitochondrial DNA (ND1, ND5) and nuclear DNA (SLCO2B1, SERPINA1) genes.

### Mitochondrial membrane potential assay

TMRE-Mitochondrial membrane potential assay from Abcam (ab113852) was used to determine the membrane potential of mitochondria. 20,000 cells /well were plated in a 96-well tissue culture plate incubated in a 37˚C 5% CO_2_ incubator overnight. Cells treated with 20 µM of FCCP for 10 min was used as a positive control. The assay was performed according to the manufacturer’s protocol.

### Cholesterol and triglyceride quantification

Cholesterol and triglyceride were quantified in harvested samples (10^6^ cells) from control and infected cells using Abcam HDL and LDL/VLDL Cholesterol assay kit (ab65390) and Abcam Triglyceride quantification assay Kit (ab178780) as per the manufacture’s protocol. 2 µl and 0.5 µl of samples were used for cholesterol and triglyceride assays in a black 96 well and a white 96 well plate with a clear bottom respectively.

### Pyruvate and lactate assays

Concentrations of pyruvate and lactate in control and infected samples were measured using the Pyruvate assay kit (ab65342) and L-Lactate assay kit (ab65331) from Abcam as per the manufacture’s protocol. 20 µl and 25 µl of the samples were used for pyruvate and lactate assay respectively and performed in a 96 well black plate with a clear bottom.

### Isolation of mitochondria

Harvested cells were washed twice with PBS and centrifuged at 276$\times$ g for 5 min. The cells were then resuspended in 1$\times$ MTE (Mannitol/Tris/EDTA) buffer consisting of 10 mM Tris base, 2 mM MgCl_2_, 270 mM D-Mannitol, 0.1 mM EDTA, 1 mM Diothiothreitol (DTT), 1$\times$ protease inhibitor cocktail (Catalogue # 11836170001) and subjected to sonication in a Branson Digital Sonifier at an amplitude of 35% for 15 sec, consisting of three 5 sec on and off intervals. The suspension was centrifuged at 1462 $\times$ g for 5 min at 4 ˚C, then the supernatant was transferred to a microfuge tube and centrifuged at 13,000$\times$ g for 10 min at 4 ˚C to obtain a pellet. 5 ml of 1.8 M sucrose was added to an ultracentrifuge tube followed by 1 M sucrose. The pellet was resuspended in 1 ml MTE buffer and added on top of the 1 M sucrose. The tubes containing the cell suspension were centrifuged in an ultracentrifuge at 83,000 $\times$ g for 25 min at 4 ˚C. The white layer at the interface of the sucrose solutions was carefully removed using a syringe, diluted with MTE buffer, and centrifuged at 13,000 $\times$ g for 5 min at 4 ˚C to remove residual sucrose. The supernatant was discarded, the pellet was mixed with MTE buffer and protein estimation was performed using the Bio-Rad DC Protein Assay.

### Stable Isotype Labelling of Amino acids in Cell culture (SILAC)

HepG2 was cultured in DMEM SILAC media ( ThermoFisher Scientific, # 88364) containing medium isotopes of amino acids, L-Arginine: HCL (U-13C6, 99%) (# CLM-2265-H-0.25) and L-Lysine: 2HCL (4,4,5,5-D4, 96-98%) (# DLM-2640-0.5); HepAD38 was cultured in DMEM/F12 SILAC media (ThermoFisher Scientific, # 88370) containing heavy isotopes of the amino acids L-Arginine: HCL (U-13C6, 99%; U-15N4, 99%) (# CNLM-539-H-0.25) and L-Lysine: 2HCL (U-13C6, 99%; U-15N2, 99%) (# CNLM-291-H-0.25). 10% of dialysed foetal calf serum (a kind gift from Eileen McGowan from University of Technology Sydney) was added to the media and the cells were incubated at 37 ˚C in a 5% CO_2_ incubator and cultured over 5 passages to allow complete incorporation of the amino acid isotopes. The cells were harvested for mitochondria isolation and processed for mass spectrometry.

### Sample preparation and SDS PAGE for mass spectrometry

Equal amounts of mitochondrial protein from HepG2 and HepAD38 were mixed, SDS gel loading dye (5% β-Mercaptoethanol, 0.02% bromophenol blue, 30% glycerol, 10% SDS and 250mM Tris Cl, pH 6.8) was added and samples were loaded onto 12% SDS-PAGE gels. Electrophoresis was carried out until the dye reached ¾ of the gel length. The gel was then stained with Coomassie Blue solution and destained with 1% acetic acid solution.

### Trypsin Digestion

The bands were cut into several pieces, added to a sterile LoBind tubes (Eppendorf) and destained using 40% acetonitrile containing 50 mM ammonium bicarbonate by shaking at room temperature for 10 min. This process was repeated 2-4 times until all the dye has been removed, followed by rinsing twice with 100% acetonitrile. The gel pieces were subjected to vacuum centrifugation until dry. 15 µl of sequencing grade modified trypsin (Promega) (12 ng/ul) in 50 mM ammonium bicarbonate was added and incubated for 1 h at 4 ˚C. Excess trypsin was removed, 15 µl ammonium bicarbonate added, and samples were incubated at 37 ˚C overnight. 20-30 µl of extract solution (10% (v/v) acetonitrile, 0.5% (v/v) TFA) was added and incubated for 15 min in a water bath sonicator. All the liquid was transferred to clean tubes and subjected to vacuum centrifugation until dry. Meanwhile the gel pieces were re-extracted with 20-40 µl of extract solution, pooled with the first extract and dried. The samples were resuspended in 30 µl 0.5% (v/v) formic acid in water.

### Stage tip clean up

Using 3M Empore C18 filter membrane (3M Bioanalytical Technologies, MN, USA) and R2 material slurry in 100% acetonitrile, column was packed in a 20 µl Eppendorf GELoader tip and activated with 30 µl of 70% (v/v) acetonitrile, 0.5% formic acid. The column was equilibrated with 2 x 30 µl 0.5% formic acid and the sample was loaded. The column was washed with 2 x 30 µl 0.5% formic acid and the peptides were eluted in 10-15 µl of 70% (v/v) acetonitrile, 0.5% formic acid. The eluate was dried in a speed-vac and resuspended in 0.1% or 0.5% formic acid.

### Mass spectrometry and data analysis

Samples were analysed using liquid-chromatography mass spectrometry (LC-MS) to measure the relative abundance of mitochondrial proteins. Peptide separation was performed on a Dionex UltiMate 3000 RSLCnano system (Thermo Scientific) with an in-house prepared nanocolumn using fused silica tubing (360mm outer and 75 mm inner diameter) packed with Dr Maisch Reprosil C18 AQ 1.9 mm particles. Peptides were loaded in buffer A (0.1% formic acid) and eluted over a 60 min or 180 min multi-step gradient to buffer B (100% acetonitrile, 0.1% formic acid) at 250 nL/min. Mass spectra were acquired in data-dependent mode on an LTQ Velos Pro Orbitrap Elite mass spectrometer (Thermo Scientific) and over a mass range of 550-2400 m/z, with automatic switch between MS and MS/MS using the top 15 method. The data were acquired and analysed at Biomedical Proteomics (cmri.org.au/Biomedical-Proteomics), Children's Medical Research Institute, Westmead.

Proteome Discoverer version 2.2 was used for data analysis, specifying MASCOT for search engine with the appropriate species specific FASTA database from Uniprot, and Percolator for false discovery rate (FDR) calculations which was set at <1%. Database for Annotation, Visualization, and Integrated Discovery (DAVID version 6.8) was used to identify enriched annotation terms associated with Gene Ontology and to cluster the functional annotation terms.

### GEO Dataset analysis

To assess clinical impact of our findings, we analysed available online datasets of human biopsy samples to examine the effects of HBV or HCV infection on key liver genes. For HBV datasets GSE66698, GSE83148 and GSE84044 were analysed. For HCV datasets GSE15331, GSE15654 and GSE34798 were analysed.

For gene expression array analysis, public array data analysis was performed in the R statistical environment (V4.2, R Core Team (2021). R: A language and environment for statistical computing. R Foundation for Statistical Computing, Vienna, Austria. URL <https://www.R-project.org/>.) on data accessed using GEOquery [2]. Normalized log-intensity expression levels were compared between sample groups by anova and plotted in tidyverse (Alboukadel Kassambara (2020). ggpubr: 'ggplot2' Based Publication Ready Plots. R package version 0.4.0. https://CRAN.R project.org/package=ggpubr) [3]._ggprism: A ‘ggplot2’ Extension Inspired by ‘GraphPad Prism’_.R package version 1.0.4, <https://CRAN.R-project.org/package=ggprism>).

## REFERENCES

1. Wakita T, Pietschmann T, Kato T, et al. Production of infectious hepatitis C virus in tissue culture from a cloned viral genome. Nat Med **2005**; 11:791-6.

2. Davis S, Meltzer PS. GEOquery: a bridge between the Gene Expression Omnibus (GEO) and BioConductor. Bioinformatics **2007**; 23:1846-7.

3. Wickham H, Averick M, Bryan J, et al. Welcome to the Tidyverse. J Open Source Softw **2019**; 4.
